# Antiviral Potential of *Chiococca alba* (L.) Hitchc. Plant Extracts Against *Chikungunya* and *Mayaro* Viruses

**DOI:** 10.3390/ijms252111397

**Published:** 2024-10-23

**Authors:** Ellen Caroline Feitoza Pires, Francini Pereira da Silva, Karoline Schallenberger, Bruna Saraiva Hermann, Larissa Mallmann, Wellington Souza Moura, Sergio Donizeti Ascêncio, Robson dos Santos Barbosa, Ilsamar Mendes Soares, Juliane Deise Fleck, Eugênio Eduardo de Oliveira, Guy Smagghe, Bergmann Morais Ribeiro, Raimundo Wagner de Souza Aguiar

**Affiliations:** 1 Department of Biotechnology, Molecular Biology Laboratory, Federal University of Tocantins, Gurupi 77410-570, Brazil; ellen.pires@aluno.unb.br (E.C.F.P.); rwsa@uft.edu.br (R.W.d.S.A.); 2 Department of Cell Biology, Institute of Biology, University of Brasília, Brasília 70910-900, Brazil; 3 Institute of Health Sciences, Molecular Microbiology Laboratory, Feevale University, Novo Hamburgo 93525-075, Braziljulianefleck@feevale.br (J.D.F.); 4 Department of Biotechnology Biodiversity and Graduate School of Biotechnology of Amazônia (Bionorte), Natural Products Laboratory, Federal University of Tocantins, Gurupi 77410-570, Brazil; bussund@gmail.com (W.S.M.); sergioda@uft.edu.br (S.D.A.); robson.barbosa@uft.edu.br (R.d.S.B.);; 5 Departamento de Entomologia, Universidade Federal de Viçosa, Viçosa 36570-900, Brazil; eugenio@ufv.br; 6 Department of Plants and Crops, Ghent University, 9000 Ghent, Belgium; 7 Institute of Entomology, Guizhou University, Guiyang 550025, China; 8 Department of Biology, Vrije Universiteit Brussel, 1050 Brussels, Belgium

**Keywords:** cainca plants, flavonoids, *Alphavirus*, viral infections, nsP2 protease

## Abstract

Chikungunya and Mayaro fevers are viral infectious diseases characterized by fever and arthralgia, for which there are currently no effective vaccines or treatments. The urgent need for novel antiviral agents against Chikungunya virus (CHIKV) and Mayaro virus (MAYV) has led to interest in plant-based compounds that can disrupt the viral replication cycle. *Chiococca alba* (L.) Hitchc., a Neotropical plant traditionally used by Yucatec Maya healers as an antipyretic and antirheumatic, may hold potential as a source of antiviral agents. This study aimed to evaluate the antiviral potential of *C. alba* methanolic extracts (CAH21 and CAH24) against CHIKV and MAYV through preliminary in vitro and in silico analyses. The cytotoxicity of two methanolic extracts from *C. alba* roots was assessed in Vero cells using the neutral red assay, and their viral activity was determined via plaque assay post-treatment. Given the observed antiviral effects, we used computational predictions to explore interactions between the multifunctional nsP2 proteases and secondary metabolites identified in *C. alba* extracts. The metabolites were identified using high-performance liquid chromatography (HPLC) and gas chromatography–mass spectrometry (GC-MS). Phytochemical analysis revealed the presence of flavonoids, coumarins, and phenolic acids in the *C. alba* extracts. In vitro assays demonstrated that both extracts inhibited over 70% of activity against CHIKV and MAYV at a concentration of 60 µg/mL. In silico predictions suggested that the flavonoids naringin and vitexin had the highest affinity for the nsP2 proteases of CHIKV and MAYV, indicating their potential as viral inhibitors. Our findings revealed that *C. alba* extract represents a promising source of novel antiviral compounds.

## 1. Introduction

Arbovirus-borne diseases pose significant public health challenges in many countries within the Neotropical region, frequently leading to outbreaks and epidemics [1]. Chikungunya virus (CHIKV) and Mayaro virus (MAYV) belong to the Togaviridae family and the Alphavirus genus. Both viruses are transmitted by the female mosquito of the genus Aedes, and their clinical manifestations resemble dengue, including fever, headache, and arthralgia [2,3,4]. Alphaviruses infect cells through receptor-mediated endocytosis [5]. Their genomes contain two open reading frames (ORFs): the first encodes four non-structural proteins (nsP1, nsP2, nsP3, and nsP4), while the second encodes structural proteins essential for viral assembly, including capsid (C), envelope glycoproteins (E1 and E2), and two cleavage products (E3 and 6k) [6,7]. The non-structural proteins play a crucial role in viral replication and transcription, making them prime targets for the development of antiviral drugs. Notably, nsP2 is a multifunctional protein with proteolytic activity essential for viral replication and propagation. It also facilitates the shutdown of viral gene expression when translocated to the nucleus and exhibits helicase activity, among other functions [8,9]. Additionally, nsP1 and nsP2 catalyze the capping of the negative RNA strand, while nsP3 and nsP4 are involved in replication and polymerase activities. Plant-derived compounds have been extensively studied for their antiviral properties [10,11]. Among these, secondary metabolites from various plant species have shown significant antiviral potential against multiple viruses, such as influenza, human immunodeficiency virus (HIV), herpes simplex virus (HSV), hepatitis, and coxsackievirus infections [12]. Examples of such metabolites include alkaloids, saponins, flavonoids, and coumarins [13]. These metabolites must exhibit antiviral activity and low cytotoxicity to host cells to be considered antiviral. Flavonoids, in particular, have garnered attention for their antiviral efficacy [14]. The Rubiaceae family, known for producing pharmacologically active secondary metabolites, such as flavonoids, alkaloids, and saponins [15,16,17], includes *Chiococca alba* (L.) Hitchc., an endemic shrub that can reach a height of 6 m; it is found in the American continent and is commonly known in Brazil as “Cainca” [18,19]. Traditional healers of the Yucatec Maya in southern Belize, Central America, have used the infusion of *C. alba* roots to treat fever, colds, muscle pains, rheumatism, asthma, and inflammations and as antimicrobial and insecticidal/repellent agents [20,21,22,23,24]. 

Considering the symptoms of intermittent fever caused by CHIKV and MAYV infection and the ethnobotanical knowledge of the Yucatec Maya, this study aimed to evaluate the efficacy of methanolic extracts from *C. alba* roots as a potential antiviral agent against CHIKV and MAYV in vitro using Vero cells. Additionally, we analyzed the in silico interactions between flavonoids (naringin and vitexin), which were identified in the *C. alba* extract, and the CHIKV and MAYV nsP2 proteases.

## 2. Results

### 2.1. Total Chemical Profiles of *C. alba* CAH21 and CAH24 Extracts

The composition and identification of the compounds present in the methanolic root extracts of *C. alba* are shown in Table S1, and the GC-MS chromatograms are shown in Figures S1 and S2. The GC-MS revealed 47 compounds in each extract (CAH21 and CAH24). The major compounds identified were cyclohexane, terpene, and furan class members. They were 1,2,3,5-cyclohexanetetrol (15.85%), cis-valerenyl acetate (12.77%), and 5-hydroxymethylfurfural (9.52%), respectively. Additionally, two compounds were identified in two peaks with different retention times: peaks 41 and 43, and peaks 37 and 44 (PubChem CID: 608286, 91730075).

### 2.2. Partial Chemical Profiles of *C. alba* CAH21 and CAH24 Extracts

High-performance liquid chromatography (HPLC) analysis revealed 28 peaks in the methanolic extract by maceration (CAH21) (Figure S3) and 21 peaks in the methanolic extract by Soxhlet (CAH24) (Figure S4). Using the available standards, the CAH21 extract was found to contain the flavonoid naringin. The CAH24 extract showed the presence of syringic acid, chlorogenic acid, and the flavonoids vitexin, myricetin, and quercetin (Table 1).

### 2.3. Cytotoxic Assay

**Figure 1:**
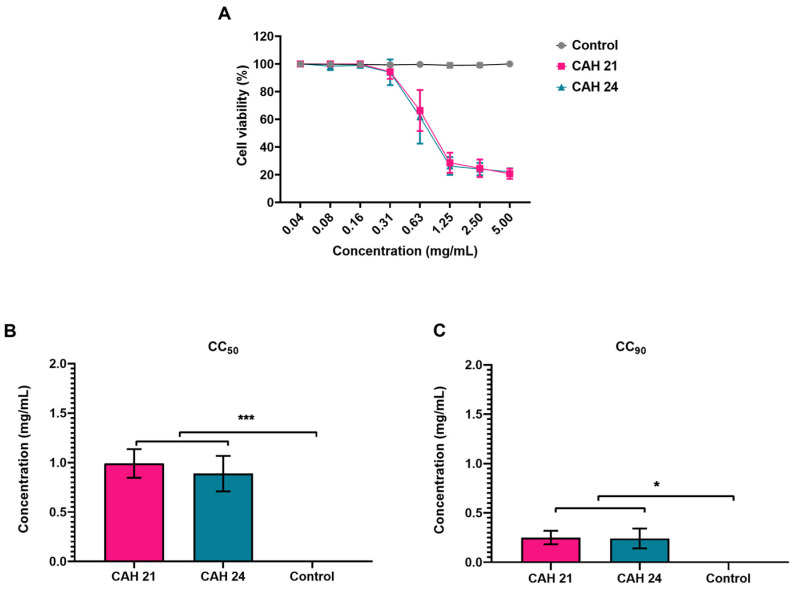
Cytotoxicity assay. (**A**) Cell viability after treatment using Vero cells, incubated for 48 h with different concentrations of *C. alba* extracts CAH21 and CAH24. (**B**) CC_50_ and (**C**) CC_90_ values. Ordinary one-way ANOVA multiple comparisons were used to determine statistical significance from cell control (* *p* = 0.1; *** *p* < 0.001).

CAH21 and CAH24 *C. alba* extracts were incubated with Vero cells for 48 h, and the CC_50_ and CC_90_ values were calculated using nonlinear regression (curve fit). They exhibited comparable cytotoxicity in Vero cells after 48 h (Figure 1A). The CC_50_ values were 0.991 ± 0.0014 mg/mL and 0.887 ± 0.0012 mg/mL for CAH21 and CAH24, respectively (Figure 1B), while the CC_90_ values were 0.260 ± 0.0001 mg/mL and 0.241 ± 0.0001 mg/mL, respectively (Figure 1C). The observed cytotoxic effects included the formation of vacuoles and cell lysis.

### 2.4. Antiviral Activity Analysis

**Figure 2:**
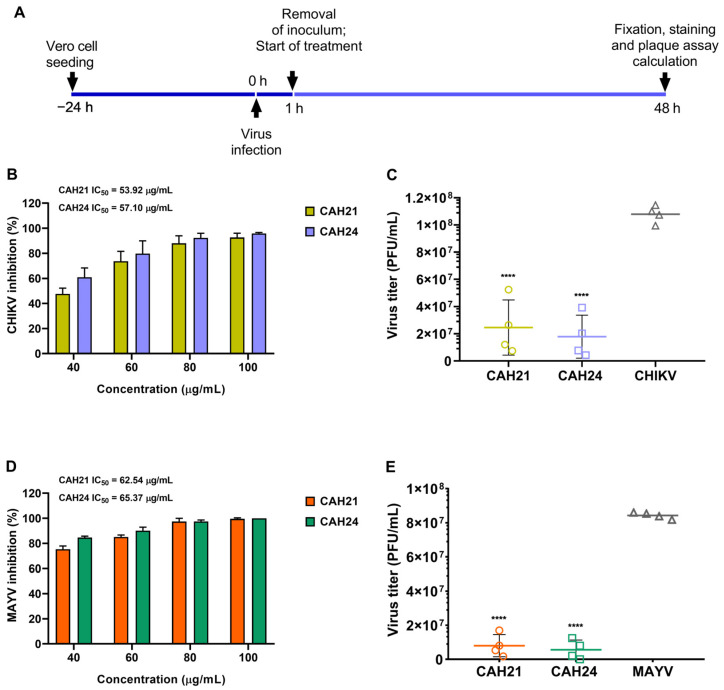
Antiviral activity of *C. alba* extracts against CHIKV and MAYV. (**A**) Schematic description of the antiviral assay. (**B**) Dose–response antiviral activity of *C. alba* extracts on the replication of CHIKV in a plaque reduction assay. The data are the means and standard deviations of three independent experiments. (**C**) The scatter dot plot shows the individual and mean values of CHIKV titer (log_10_ PFU/mL) at 40, 60, 80, and 100 μg/mL of *C. alba* extracts. (**D**) Dose–response antiviral activity of *C. alba* extracts on the replication of MAYV in a plaque reduction assay. The data are the means and standard deviations of three independent experiments. (**E**) The scatter dot plot shows the individual and mean values of the MAYV titer (log_10_ PFU/mL) at 40, 60, 80, and 100 μg/mL of *C. alba* extracts. Inhibitory concentration 50% (IC_50_) values were calculated by nonlinear regression analysis using GraphPad Prism software (version 8.01) by plotting log inhibitor versus normalized response (variable slope). An ordinary one-way ANOVA with multiple comparisons was used to determine statistical significance from viral control (**** *p* < 0.0001).

The antiviral assay was performed in the post-treatment step, as illustrated in Figure 2A (see Figures S5 and S6). Upon comparison, CAH21 exhibited a lower concentration for the inhibition of 50% of the virus replication for both viruses tested (see Figure 2B,D). Specifically, the reduction in CHIKV plaque formation exceeded 70% at a concentration of 60 μg/mL for both extracts. Additionally, CAH21 at a concentration of 40 μg/mL reduced plaque formation by 47% (Figure 2B).

The antiviral assays with the CAH24 extract exhibited a greater reduction in plaque formation compared to CAH21 (Figure 2C,E). Specifically, for the CAH24 extract, 84% and 90% inhibition of MAYV replication were observed at concentrations of 40 μg/mL and 60 μg/mL, respectively (Figure 2D). Therefore, both the CAH21 and CAH24 extracts demonstrate potential anti-MAYV and anti-CHIKV activities (Table 2).

### 2.5. Modeling and Validation of the MAYV nsP2 Structure 

**Figure 3:**
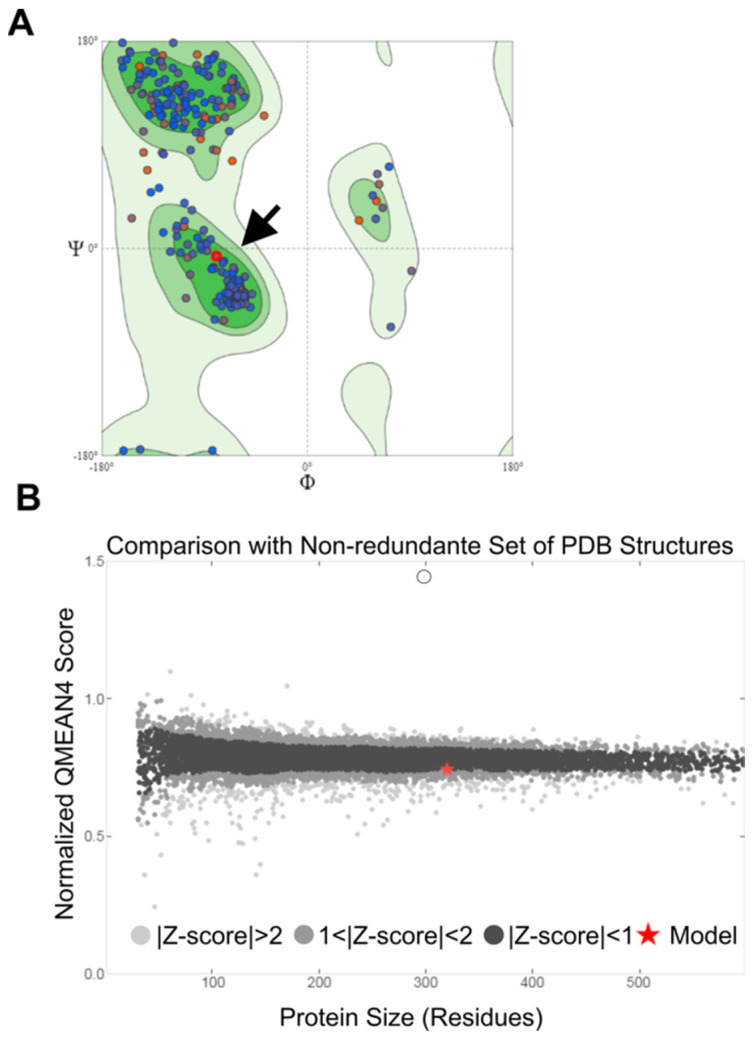
MAYV nsP2 structure modeling and validation. (**A**) Ramachandran’s graph for MAYV nsP2. The black arrow indicates the presence of the protein in the most favorable region. Dots show where the dihedral angles of amino acids in a protein structure fall. Blue dots represent residues that adopt allowed or stable conformations according to the standard geometry of proteins, while red dots highlight residues with unusual or less favorable dihedral angles. (**B**) The QMEAN (qualitative model energy analysis) value, which describes the quality of the model. The red star indicates that the position in Z-scores presents a highly reliable structure and is within the range of scores typically found for proteins of similar size.

The three-dimensional structure of the MAYV nsP2 was modeled using the CHIKV nsP2 as a template (PBD ID: 4ZTB). To ensure the adequacy of the model construction, the amino acid sequences must exhibit highly similar structures, as significant disparities could compromise the functionality of the model. Ideally, this identity should exceed 30% [25,26], and we found a 67.50% identity between the model used and the protein’s three-dimensional structure. Upon analysis of the Ramachandran plot, we observed that the protein structure was energetically favorable with 93.08% and a value close to zero (−0.79) for the QMEAN analysis (Figure 3A,B), indicating the good precision and quality of the model [27,28].

### 2.6. Molecular Docking

According to the in silico analysis, the molecules identified in the *C. alba* extracts can interact with the CHIKV and MAYV nsP2 proteins, exhibiting varying affinity energies, as detailed in Table 3. Notably, naringin and vitexin displayed strong interactions with nsP2, indicating their potential anti-CHIKV and anti-MAYV molecules, based on the best interaction energies between the molecule and receptor [29]. Molecular docking using naringin and vitexin with the nsP2 of CHIKV and MAYV was conducted to evaluate the antiviral potential of these flavonoids. This assessment was based on the hypothesis that flavonoids might disrupt the viral replication cycle, given that the post-treatment step was employed in the plaque reduction assay.

**Figure 4:**
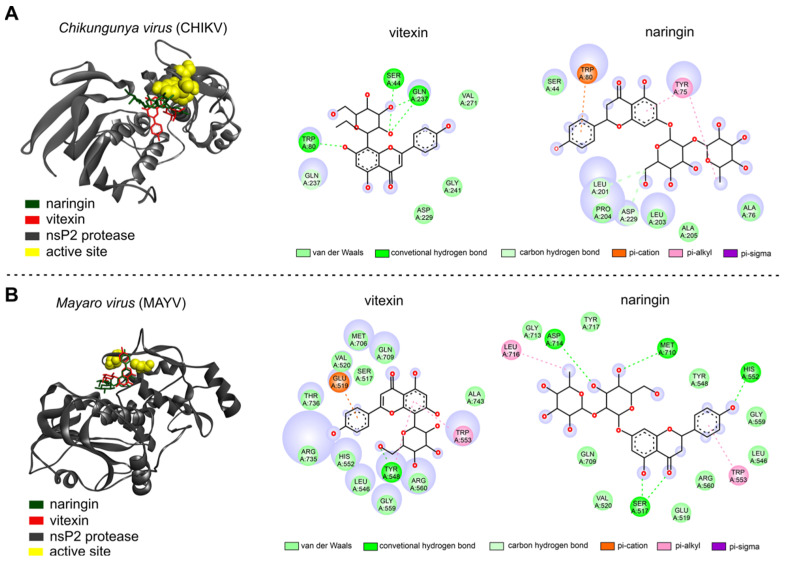
Molecular docking. (**A**) Complex between the nsP2 proteases of CHIKV and (**B**) MAYV with molecules of ligand naringin (green) and vitexin (red), close to the region of the active site (yellow), and a 2D interaction map with the amino acids.

Initially, the nsP2 protease of CHIKV was targeted. Vitexin showed hydrogen bond interactions with the nsP2 active site, specifically with SER44, GLN237, and TRP80. On the other hand, the naringin–CHIKV–nsP2 complex exhibited van der Waals interactions with amino acids such as SER44, PRO204, LEU203, ALA205, and ALA76. Additionally, it displayed one pi-alkyl interaction, one pi-sigma interaction with TYR75, hydrogen carbon interactions with LEU201 and ASP229, and one pi-cation interaction with TRP80 (Figure 4A). Subsequently, the MAYV nsP2 protease was targeted. In the vitexin–MAYV–nsP2 complex, van der Waals interactions were observed with amino acids such as MET706, GLN709, SER517, VAL520, THR736, ARG735, HIS552, LEU546, GLY559, ARG560, and ALA743. Furthermore, a carbon-hydrogen interaction with TYR548, a pi-alkyl interaction with TRP553, and a pi-cation interaction with GLU519 were observed. On the other hand, the naringin–MAYV–nsP2 complex demonstrated a total of 15 interactions, including van der Waals bonds with TYR717, GLY713, GLN709, VAL520, GLU519, ARG560, LEU546, GLY559, and TYR548. Additionally, it showed carbon-hydrogen interactions with ASP714, MET710, HIS552, and SER517, as well as pi-alkyl interactions with TRP553 and LEU716 (Figure 4B).

## 3. Discussion

Here, we demonstrated the anti-CHIKV and anti-MAYV activities of *C. alba* methanolic plant extracts. The cytotoxicity of *C. alba* extracts to Vero cells was assessed, and a concentration–response curve was established. An antiviral drug needs to exhibit activity against the virus without inducing significant toxicity to the host cell. Therefore, investigating cytotoxicity activity is the initial step in antiviral testing. Given the crude nature of the tested extracts, the observed cytotoxic effects may be attributed to the presence and concentrations of the molecules therein, as the stock solutions were prepared without organic solvents. Higher concentrations of metabolites could lead to increased cytotoxicity. Additionally, the in silico analyses revealed that some of these compounds interacted more effectively with the nsP2 protease of CHIKV and MAYV, suggesting a potential antiviral mechanism.

An extract exhibiting potential antiviral activity typically demonstrates a reduction in plaque formation ranging between 50 and 90% compared to controls. To be classified as antiviral, an extract should achieve a reduction of >90% [30]. The methanolic extract obtained using the Soxhlet, CAH24, displayed superior antiviral activity against CHIKV and MAYV compared to the maceration-derived CAH21 extract but was not statistically significant. The Soxhlet extraction system maintains high temperatures throughout the process, promoting increased solubility and diffusivity of the sample [31]. Consequently, the high temperature may have allowed the obtention of antiviral metabolites in higher concentrations, though they resulted in a lower CC_50_ when compared to the CAH21 extract.

Partial analysis via the HPLC of the *C. alba* extracts revealed 28 peaks for CAH21 and 21 for CAH24. However, due to the utilization of only 16 known standard molecules, it was possible to identify only six metabolites: chlorogenic acid, naringin, syringic acid, vitexin, myricetin, and quercetin. Therefore, GC-MS analysis was employed to identify additional compounds in *C. alba*, which included esters, fatty acids, alcohols, aldehydes, and terpenes. The results showed that GC-MS offered a simple, rapid, and susceptible method for component analysis in *C. alba* roots. The different extraction and preparation methods of the *C. alba* extracts enabled the evaluation and comparison of the profiles obtained in the cytotoxicity and antiviral tests.

We searched the literature for the three major compounds (1,2,3,5-cyclohexanetetrol, cis-valerenyl acetate, and 5-hydroxymethylfurfural) that GC-MS identified. Researchers identified the compound 1,2,3,5-cyclohexanetetrol in the hydroethanolic extract of *A. paniculata* leaves (15.10%) and in the ethanolic extract of *Clitoria ternatea* leaves (3.55%). And the studies reveal that these extracts have cardioprotective action in isoproterenol-induced myocardial infarction and nephroprotective action in acetaminophen-induced toxicity in mice, respectively. There is no data regarding the antiviral activity of 1,2,3,5-cyclohexanetetrol [32,33]. The terpene molecule, cis-valerenyl acetate, was found in oils from the roots and leaves of *Valeriana pyrolaefolia* Decne. Other investigations performed phytochemical analysis but did not test for in vivo, in vitro, and in silico activity. There is no investigation of the antiviral activity of cis-valerenyl acetate in the literature [34,35].

5-Hydroxymethylfurfural (5-HMF) is an endogenous product found in plants, in free or bound forms, and is present in a range of sugar-rich foods and traditional Chinese medicines [36]. Pharmacological studies of the components showed that 5-HMF presented good biological activities, such as anti-inflammatory activity and bacteriostatic action [37]. A recent study reported, for the first time, that 5-HMF has an immunomodulatory mechanism against vesicular stomatitis virus (VSV) infection. In summary, these findings indicate that 5-HMF can induce type I IFN production and enhance IFN-JAK/STAT signaling in primary peritoneal macrophages [38].

To date, there are no published data on the antiviral activity of *C. alba* extracts against arboviruses. Since the specific compound or compounds responsible for the possible antiviral activity of the extracts are unknown, fractionating the different compounds present in the extract to test their antiviral activity is necessary. Based on our results, we hypothesize that the flavonoids—naringin and vitexin—found in *C. alba* might interact with viral replication enzymes; this mechanism is supported by the literature that describes flavonoids as having antiviral activity [14,39,40,41,42], particularly against arboviruses [43,44,45,46,47,48,49,50,51,52,53], enteroviruses [54,55], hepatitis viruses [56,57], and influenza viruses [50,58].

Other studies conducted antiviral activities using four flavonoids (quercetin, naringin, hesperetin, and daidzein) against dengue virus type 2 (DENV-2). It was demonstrated that quercetin showed an IC_50_ = 35.7 μg/mL, while naringin exhibited an IC_50_ of 168 μg/mL when used before virus adsorption by plaque assay. Daidzein displayed low activity against DENV-2, and hesperetin was not effective against DENV-2. Additionally, the presence of 50 µg/mL of quercetin reduced DENV-2 RNA production levels by 67% compared to the control [48]. Naringenin and quercetin flavonoids have also shown antiviral activity against DENV-1 to 4 and MAYV, respectively [51,52]. In another study, the authors screened methanolic extracts of medicinal plants for their anti-DENV activity. The species *A. paniculata* and *Momordica charantia* inhibited DENV replication by 75 and 50%, respectively. The authors attributed the antiviral action to the flavonoids kaempferol, quercetin (found in *M. charantia*), and luteolin [53].

In our study, two of these flavonoids were identified by HPLC: naringin in the CAH21 extract and quercetin in the CAH24 extract. Syringic acid identified in CAH24 has not previously been correlated with antiviral activities against arboviruses, and there are limited studies on the antiviral activity of chlorogenic acid [58]. However, it has been suggested that the antiviral activity (against herpes simplex virus, HSV) of substances isolated from *Persea americana* may be due to a synergism between flavonoids and chlorogenic acid [56]. 

The flavonoids’ antiviral activity has been linked to the inhibition of viral RNA polymerase, as well as the prevention of viral adhesion and entry into cells [43,46,59]. The affinity between the constituents present in the extract and specific viral proteins largely depends on the types of chemical bonds formed between these substances and the amino acids of the viral proteins. The separation of nonpolar and polar components from plant extracts may increase the likelihood of discovering highly active antiviral compounds with low cytotoxicity [39]. Therefore, elucidating the antiviral mechanism of *C. alba* extracts necessitates the identification of their biologically active constituents, which would allow the active principle to be used without interference from other metabolites. The potential antiviral activity found in the *C. alba* extracts can likely be attributed to the presence of major components, such as flavonoids.

In this study, we conducted an in silico analysis of the potential binding of the metabolites identified in the *C. alba* extracts with the nsP2 protease of CHIKV and MAYV. Given their antiviral activity, the flavonoid molecules detected in both extracts were analyzed for their interactions with the nsP2 proteins of CHIKV and MAYV. Beyond its protease activity, nsP2 also exhibits other enzymatic functions during viral replication, such as RNA helicase and RNA triphosphatase activities, which are responsible for unwinding duplex RNA and removing the phosphate-5’ group from viral RNA during the capping reaction [7]. Molecular docking analysis revealed that the most common type of interactions in the active sites of nsP2 for both CHIKV and MAYV were van der Waals interactions. Although the van der Waals interactions are generally considered weak, their prevalence suggests a good correlation between the protein binding site and the ligand structure, potentially leading to effective antiviral activity [60,61].

The high-resolution crystal structure of CHIKV nsP2 facilitates the search for antiviral molecules. To construct the crystal structure of MAYV nsP2, the Ramachandran plot is a critical quality assessment tool for selecting experimental structure models. The Ramachandran values indicate the distribution of protein torsion angles (φ, Ψ), with an ideal reference parameter close to 96% [28,61,62]. 

Recently, the flavanone glycoside naringin was identified as binding to nsP2 protease with nanomolar affinity through a combination of receptor-based docking and molecular dynamics (MD) simulations. The protease–naringin complex was subjected to MD simulations to test the stability of the protein–ligand complex. Using the ParDOCK server, it was found that naringin had a strong affinity for the nsP2 active site, with an affinity energy of −9.47 kcal/mol, supporting our findings [62]. Additionally, an in silico study examining the possible molecular interactions between CHIKV nsP3 and three potential flavonoid ligands was conducted. Among these, the flavonoid baicalin showed the highest binding affinity (−9.8 kcal/mol), suggesting it could be a potent CHIKV nsP3 inhibitor [60]. 

To our knowledge, this is the first study demonstrating the in silico interaction between flavonoids and MAYV nsP2 protease. However, the antiviral activity of the flavonoid epicatechin isolated from *Salacia crassifolia* against the MAYV capsid protein (protein C) was identified previously [42]. Additionally, the anti-MAYV and anti-CHIKV activities of the flavonoid silymarin have been confirmed through in vitro biological assays [63,64]. 

Natural products, whether in their native form or after extraction of their active ingredients, have long been used by different populations. The use of plants in traditional Brazilian medicine to treat various diseases is increasing, due to the diversity of the bioactive constituents they contain, making them potential sources of antiviral substances [10,11,12,13,14]. Traditional ethnopharmacology has enabled, through isolation and characterization methods and the development of specific chemoinformatics methods, the discovery of many modern drugs, such as aspirin, atropine, ephedrine, digoxin, morphine, quinine, reserpine, and tubocurarine. These are some examples of the drugs that were originally discovered through the study of the traditional cures and popular knowledge of indigenous peoples [65]. The knowledge of ethnopharmacology, supported by its experience-based holistic and systemic approach, can serve as an innovative and powerful discovery mechanism for newer, safer, and more accessible drugs [66]. In this context, the prospect of discovering new plant-derived drugs remains highly relevant and is supported by numerous publications demonstrating the antiviral activity of plant derivatives [10], their broad structural diversity, and their extensive availability in nature [11].

## 4. Materials and Methods

### 4.1. Plant Material

*C. alba* roots were collected in March 2019 in Formoso do Araguaia (11°47′48″ S latitude, 49°31′44″ W longitude), Tocantins state, Brazil [24]. The plant was identified at the herbarium of the Department of Environmental Studies at the Federal University of Tocantins (Campus Porto Nacional), where a specimen voucher was deposited under the code HTO-11.160. The research was authorized by the Sistema Nacional de Gestão do Patrimônio Genético e do Conhecimento Tradicional Associado (SISGEN) under the number A77A809.

### 4.2. Preparation of Extracts

The plant material powder was subjected to two different extraction processes: maceration (CAH21) and Soxhlet extraction (CAH24), using a Marconi, model MA-487/6/25, (São Paulo, Brazil). For the CAH21 process, powdered roots (500 g) were extracted at room temperature (25 °C) for three days with methanol (MeOH). For the CAH24 process, powdered roots (500 g) were extracted at 60 °C for 4 h with MeOH. The samples were filtered through Whatman No. 1 filter paper (GE Healthcare Life Sciences, Chicago, IL, USA), and the liquid fractions were concentrated using a rotary evaporator to obtain crude extracts [24,67]. Each methanol extract was lyophilized and stored in a moisture-free desiccator until use. Stock solutions were prepared at a concentration of 10,000 μg/mL, using only Dulbecco’s modified Eagle medium (DMEM) (Cultilab, São Paulo, Brazil), with no organic solvents used to solubilize the extracts. Before the assays, the fractions were filtered using a Durapore^®^ membrane filter, 0.22 μm.

### 4.3. Chemical Analysis of Extracts

The chemical profiles of the *C. alba* root extracts (CAH21 and CAH24) were analyzed in triplicate using a gas chromatograph (Shimadzu, model QP2020, Tokyo, Japan) coupled to a mass spectrometer (GC-MS) at the Analytical Center of the Institute of Chemistry of the University of São Paulo (IQUSP). Separation of the target analytes was achieved on a DB-1 capillary column (30 m × 0.25 mm i.d., 0.25 μm film thickness). The analysis conditions were as follows: Helium was used as the carrier gas at a flow rate of 2.5 mL/min; the temperature program ranged from 50 °C to 280 °C at a rate of 60 °C/min; the injector and interface temperatures were maintained at 280 °C; and the mass spectrometer operated with an electron impact ionization of 70 eV and swept from 50 to 700 μs. Compounds were identified by comparing their spectra with the National Institute of Standards and Technology (NIST) database. The structures of the components in the test materials were identified based on their molecular weights.

The partial chemical profiles of the *C. alba* extracts (CAH21 and CAH24) were analyzed by high-performance liquid chromatography (HPLC) at the Scientific Instrumentation Laboratory (LABIC) of the Federal University of Tocantins. The analysis was conducted using a SHIMADZU^®^ HPLC system (Kyoto, Japan) consisting of a LC-10ATVp pump, CTO-10A column oven, DGU14A degasser, SCL 10A system controller, and Shimadzu SPD-10AT UV-VIS detector, with a loop injector with a 20 μL loop size. 

Chromatographic analysis was performed under gradient conditions using a C-18 reverse-phase column (250 × 4.6 mm, particle size 5 μm, Luna 5 μ C-18). The elution solvents were 0.1% phosphoric acid in water (solvent A) and 0.1% phosphoric acid in water/acetonitrile/methanol (54:35:11 *v*/*v*) (solvent B) (Table S2). The flow rate was set at 1.0 mL/min, with a detection wavelength was 280 nm. The compounds were identified by comparing sample retention times with those of the 16 available authentic standards (Figure S7) (Sigma–Aldrich, St. Louis, MO, USA). 

### 4.4. Cells and Viruses

Vero cells (ATCC^®^: CCL81)—derived from African green monkey kidney (*Cercopithecus aethiops*)—were obtained from the Feevale University Molecular Microbiology stock (Rio Grande do Sul, Brazil). These cells were cultured in DMEM supplemented with 10% fetal bovine serum (FBS) (Cultilab, São Paulo, Brazil), and maintained at 37 °C in a 5% CO_2_ atmosphere.

The viral isolates used in the experiments were a CHIKV isolate from the East/Central/South African genotype (GenBank accession no. LR2006_OPY1) and the MAYV BeAr 20290 (GenBank accession no. KT754168). The Vero cells were infected with a multiplicity of infection (MOI) = 0.01, in a 75 cm^2^ culture flask containing a monolayer of 8.5 × 10^6^ cells. The infected cells were incubated for 48 h at 37 °C in a 5% CO_2_ atmosphere. The supernatant was collected and stored at −80 °C until viral titration [67,68].

### 4.5. Viral Titration by Plaque Assay

The Vero cells were plated in 12- and 24-well plates 24 h before conducting the assay. A semi-solid medium was prepared with 2X minimum essential medium (MEM) (Cultilab, São Paulo, Brazil), and carboxymethylcellulose sodium salt (CMC) high viscosity (Sigma–Aldrich, St. Louis, MO, USA) at 1.5% for CHIKV and 1% for the MAYV titration. The culture medium in the plates was removed by aspiration, and virus inoculum of various concentrations were added to duplicate wells. The inoculum fluid was distributed by gently rocking the plates manually. The plates were then incubated at 37 °C for about 1 h, rocking every 15 to 20 min. After incubation, the plates were incubated for 5 days. For cell fixation, 4% formaldehyde was added for 30 min, and the wells were washed with 1X phosphate-buffered saline (PBS, 137.0 mM NaCl, 2.7 mM KCl, 10.0 mM Na_2_HPO_4_, 2.0 mM KH_2_PO_4_, pH 7.4) [63,68]. The plates were then stained with 0.2% crystal violet (Sigma–Aldrich, St. Louis, MO, USA) for 30 min. After staining, the crystal violet was removed, and the plaques were counted 24 h later. The viral titer was calculated in plaque-forming units (PFU/mL).

### 4.6. Cytotoxicity Assay

The cytotoxicity of the plant extracts (CAH21 and CAH24) was assessed using the standardized neutral red incorporation assay for lysosomal viability at the Department of Cell Biology, University of Brasília [69]. For this assay, 96-well plate microplates were prepared 24 h in advance, with Vero cells at a concentration of 1 × 10^5^ cells per well. After removing the medium, 150 μL of each extract’s serial dilution (starting at 5 mg/mL) was added in triplicate. Untreated cells served as controls, with an equal volume (150 μL) of DMEM added. 

The plates were incubated at 37 °C in 5% CO_2_ for 48 h. Cell viability was determined by comparing the absorbance values obtained for each concentration with those obtained for the control cells (considered 100% viable). The extract concentration toxic to 50% (CC_50_) and 90% (CC_90_) of the cells was calculated using the dose–response curve. The experiment was performed in triplicate with three independent replications.

### 4.7. Antiviral Assays

For the antiviral activity tests, microplates were prepared as previously described. After incubation at 37 °C in a humidified environment with 5% CO_2_, the viral suspension (MOI = 0.01) was inoculated. DMEM was added to the wells designated for cell control and cytotoxicity control. After 1 h of incubation with gentle shaking every 15 min, the respective viral suspensions and medium were aspirated from all the wells, which were then washed with 1X PBS. Different concentrations of plant extracts (40, 60, 80, and 100 µg/mL) were prepared with the semi-solid medium and added to the respective treatment wells. The cytotoxicity control wells received the highest concentration of the extracts (100 µg/mL). The microplates were incubated at 37 °C under a 5% CO_2_ atmosphere for 48 h. 

Subsequently, the wells were fixed and stained as previously described. The analysis was conducted in duplicate, with at least three independent experiments performed, following the standardized protocol for each virus. The percentage reduction in the number of plaques was calculated using the formula *A* (%) = 100 − [(*B* × 100)/*C*], where *A* is the percentage reduction in the plaque number, *B* is the average number of plaques for the treatments, and *C* is the number of plaques in the viral control [70]. The half-maximal inhibitory concentration (IC_50_) was calculated, and the selectivity index (SI) was determined using the ratio CC_50_/IC_50._

### 4.8. In Silico Analysis of the Interaction between Molecules of *C. alba* and the nsP2 Protease of CHIKV and MAYV

The amino acid sequence of the MAYV nsP2 protease, available at GenBank ID: AZM66145.1, was used to construct the 3D structure through homology modeling using the Swiss Model Workspace platform (https://swissmodel.expasy.org/ (accessed on 4 December 2019)). This was achieved using the homologous protein nsP2 of CHIKV, available in the Protein Data Base (PDB) ID: 4ZTB. Additionally, the 3D structure file of the CHIKV nsP2 protease is available in the PDB under ID: 3TRK.

Flavonoids and phenolic acids from *C. alba* (naringin, syringic and chlorogenic acids, vitexin, myricetin, and quercetin) were modeled using Marvin Sketch 18.10 (ChemAxon, Budapest, Hungary). Molecular docking of the receptors and ligands was performed using AutoDock Tools 1.5.7 [71], following the methodology proposed by our colleagues [72]. Docking calculations were executed with AutoDock Vina, generating nine docking positions for each ligand by interacting with the target proteins and providing affinity energy values (Kcal/mol). The docking results were analyzed using PyMOL 2.0 [73] and Discovery Studio 4.5 [74] to identify the best binding location for each ligand within the protein target.

### 4.9. Statistical Analysis

All the data were graphed using GraphPad Prism^®^ software (version 8.0.1, San Diego, CA, USA). Statistical significance was determined by ANOVA with Tukey’s multiple comparisons test, except for the cell viability studies, which utilized nonlinear regression curve fit analysis. The toxic concentration for the cells was determined from the linear equation. For all analyses, *p* < 0.05 was considered statistically significant.

## 5. Conclusions

The methanolic extracts of *C. alba* roots demonstrated potential activity against CHIKV and MAYV. In in silico analysis, they revealed that the flavonoids naringin and vitexin exhibited high binding affinity with the nsP2 protease enzymes of CHIKV and MAYV. Consequently, it is hypothesized that these bioligands may inhibit viral replication. To confirm this potential inhibition, additional studies on enzymatic activity are required, in addition to fractionation of the crude extract by guided bioassay, to isolate the antiviral molecule. Further studies with the aerial parts of the plant will be important to search for antiviral molecules that can maintain the preservation of the species in phytochemical screening research. Therefore, *C. alba* extracts represent a possible source of antiviral compounds for the development of drugs against viral infections that affect millions of people worldwide.

## Figures and Tables

**Table 1:** Chemical profile of *C. alba* extracts identified by HPLC.

CAH21								
Peak	Compound	Chemical Structure	Retention Time (min)	Molecular Formula	Molar Mass (g/mol)	Area (%)	PubChem CID	CAS Number
9	Naringin	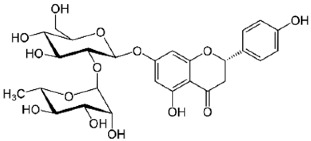	31.670	C_27_H_32_O_14_	580.54	1.28	442428	10236-47-2
**CAH24**								
5	Syringic acid	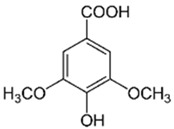	24.25	C_9_H_10_O_5_	198.17	6.27	10742	530-57-4
6	Chlorogenic acid	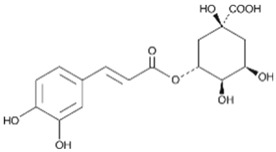	25.30	C_16_H_18_O_9_	354.31	0.53	1794427	327-97-9
10	Vitexin	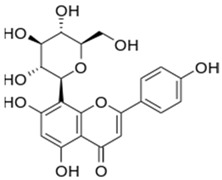	36.57	C_21_H_20_O_10_	432.38	13.01	5280441	3681-93-4
12	Myricetin	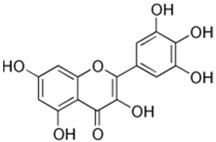	65.23	C_15_H_10_O_8_	318.23	4.13	5281672	529-44-2
14	Quercetin	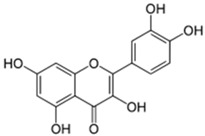	75.25	C_15_H_10_O_7_	302.23	16.86	5280343	117-39-5

Total compounds identified = 42.08%.

**Table 2:** Cytotoxicity, selectivity index, and reduction percentage in the plaque numbers for *C. alba* extracts against CHIKV and MAYV in Vero cells after 48 h.

Plant Extracts	CC_50_ (µg/mL) ^a^	CC_90_ (µg/mL) ^b^	µg/mL	CHIKV % Reduction ^c^	SI ^e^	MAYV % Reduction ^d^	SI ^e^
CAH21	991 ± 0.1	251 ± 0.1	40	47.6 ± 4.6	18.4	75.3 ± 2.6	15.8
			60	73.6 ± 8.0		85.1 ± 1.5	
			80	88.0 ± 5.9		97.4 ± 2.6	
			100	92.6 ± 3.4		99.5 ± 0.8	
CAH24	887 ± 0.2	241 ± 0.1	40	60.8 ± 7.5	15.5	84.6 ± 1.1	13.6
			60	79.8 ± 10.1		90.1 ± 2.8	
			80	92.3 ± 3.6		97.5 ± 1.2	
			100	95.81 ± 0.77		100 ± 0.01	

^a,b,c,d^ Values are mean ± SD of the triplicate assay for each sample. ^a^ CC_50_ values represent the means of three independent experiments. Statistical significance from cell control determined by ordinary one-way ANOVA multiple comparisons. ^b^ CC_90_ values represent the means of three independent experiments (*p* < 0.05). Statistical significance from cell control determined by ordinary one-way ANOVA multiple comparisons. ^c,d^ Values were calculated by nonlinear regression analysis using GraphPad Prism software (version 8.01). ^e^ SI (Selectivity Index) = CC_50_/IC_50_.

**Table 3:** Molecular docking results for the complexes formed between the *C. alba* extract molecules and the nsP2 proteins of CHIKV and MAYV.

Molecules	Receptor (kcal/mol) ^a^	
	nsP2 Protease—CHIKV	nsP2 Protease—MAYV
Chlorogenic acid	−6.7	−6.8
Myricetin	−7.0	−7.1
Naringin	−7.9	−8.5
Quercetin	−7.1	−7.3
Syringic acid	−4.6	−4.9
Vitexin	−7.9	−7.9

^a^ Affinity energy of AutoDock Vina.

## Data Availability

Data are contained within the article and Supplementary Materials.

## Supplementary Materials

The supporting information can be downloaded at: https://www.mdpi.com/article/10.3390/ijms252111397/s1.
