# Supplementary material for: Antiviral Potential of Chiococca alba (L.) Hitchc. Plant Extracts Against Chikungunya and Mayaro Viruses
Source: Int J Mol Sci. 2024 Oct 23;25(21):11397. doi: 10.3390/ijms252111397 (PMC11546558; doi:10.3390/ijms252111397)
Supplement: Supplementary file 1 [file ijms-25-11397-s001.zip › ijms-3237802-supplementary.pdf]

Supporting information

Table S1 Chemical profile of *C. alba* extracts identified by GC-MS.

Peak	Compound	Chemical structure	Retention time (min)	Molecular formula	Molar mass (g/mol)	Area (%)	PubChem CID	CAS number
1	Ethanone, 1-oxiranyl-	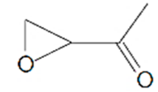	3.430	$C_4H_6O_2$	86.09	0.76	20429	4401-11-0
2	Acetic acid, butyl ester	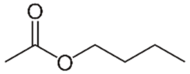	3.813	$C_6H_{12}O_2$	116.16	0.43	31272	123-86-4
3	Furfural	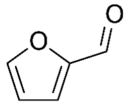	4.205	$C_5H_4O_2$	96.08	0.85	7362	98-01-1
4	Cellosolve acetate	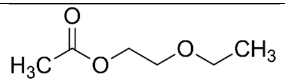	5.923	$C_6H_{12}O_3$	132.16	1.67	8095	111-15-9
5	Butane-2-one, 3-methyl-3-(2-oxopropylamino) -	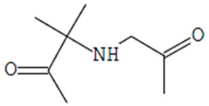	9.135	$C_8H_{15}NO_2$	157.21	0.41	575017	*
6	4H-Pyran-4-one, 2,3-dihydro-3,5-dihydroxy 6-methyl-	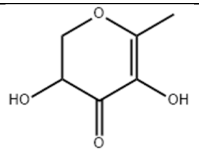	15.259	$C_6H_8O_4$	144.12	1.15	119838	28564-83-2

7	Glycolophenone	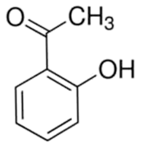	15.902	$C_8H_8O_2$	136.15	2.19	8375	118-93-4
8	5-Hydroxymethylfurfural	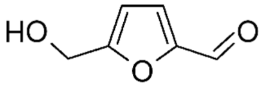	19.323	$C_6H_6O_3$	126.11	9.52	237332	67-47-0
9	4-vinylguaiacol	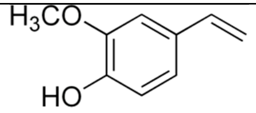	22.706	$C_9H_{10}O_2$	150.17	2.62	332	7786-61-0
10	Syringol	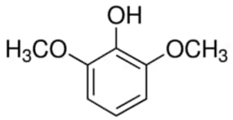	24.325	$C_8H_{10}O_3$	154.16	2.14	7041	91-10-1
11	Ethanone, 1-(2-hydroxy-5-methoxyphenyl)-	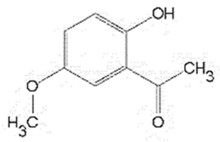	26.391	$C_9H_{10}O_3$	166.17	1.30	69714	705-15-7
12	Benzaldehyde, 2-hydroxy-4-methyl-	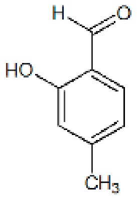	28.473	$C_8H_8O_2$	136.15	5.56	61200	698-27-1

13	3',5'-Dimethoxyacetophenone	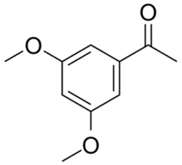	33.091	$C_{10}H_{12}O_3$	180.20	1.51	95997	39151-19-4
14	1,2,3,5-Cyclohexanetetrol	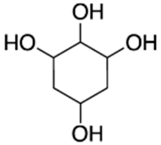	36.681	$C_6H_{12}O_4$	148.16	15.85	548226	136936-97-5
15	4-Methoxycinnamic acid	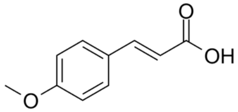	38.801	$C_{10}H_{10}O_3$	178.18	1.31	699414	943-89-5
16	Naphthalene, decahydro-1-pentadecyl-	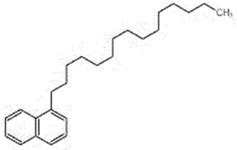	39.061	$C_{25}H_{48}$	348.6	0.49	296573	66359-82-8
17	Coniferyl aldehyde, isopropyl ether	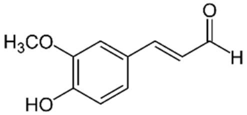	39.467	$C_{13}H_{16}O_3$	220.26	1.22	21840279	*
18	Phenol, 4-(3-hydroxy-1-propenyl)-2-methoxy	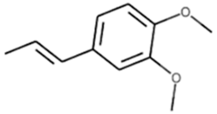	39.705	$C_{10}H_{12}O_3$	180.20	1.69	9983	458-35-5

19	3,4-Dimethoxycinnamic acid	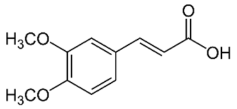	45.726	$C_{11}H_{12}O_4$	208.21	1.18	717531	14737-89-4
20	Methyl palmitate	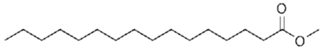	46.326	$C_{17}H_{34}O_2$	270.5	0.80	8181	112-39-0
21	Scopoletin	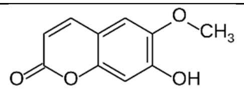	46.794	$C_{10}H_8O_4$	192.17	1.00	5280460	92-61-5
22	Palmitic acid	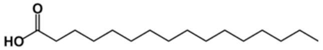	47.576	$C_{16}H_{32}O_2$	256.42	5.29	985	57-10-3
23	6,7-Dimethoxy-1,4-dihydro-2,3-quinoxalinedione	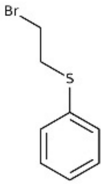	50.758	$C_{10}H_{10}N_2O_4$	222.20	1.02	613780	4784-02-5
24	Methyl lineoleate	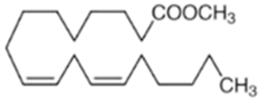	51.509	$C_{19}H_{34}O_2$	294.5	0.44	5284421	68605-14-1
25	Methyl oleate	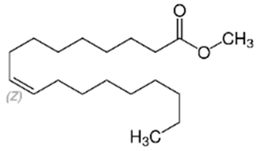	51.748	$C_{19}H_{36}O_2$	296.5	0.46	5364509	112-62-9
26	Linoleic acid	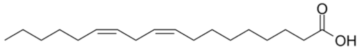	52.705	$C_{18}H_{32}O_2$	280.4	1.59	5280450	60-33-3

27	Oleic acid	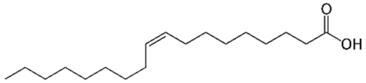	52.915	$C_{18}H_{34}O_2$	282.5	1.83	445639	112-80-1
28	Stearic acid	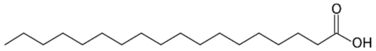	53.684	$C_{18}H_{36}O_2$	284.5	0.81	5281	57-11-4
29	Oxirane, tetradecyl-	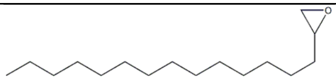	61.125	$C_{16}H_{32}O$	240.42	0.58	23741	7320-37-8
30	2-Palmitoylglycerol	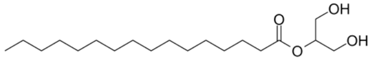	63.106	$C_{19}H_{38}O_4$	330.5	2.31	123409	23470-00-0
31	2-Naphthalenol, 2,3,4,4a,5,6,7-octahydro-1,4a-dimethyl	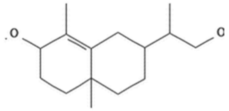	65.872	$C_{15}H_{26}O_2$	238.37	0.94	536596	*
32	10,11-Dihydro-10-hydroxy-2,3-dimethoxydibenz(b,f)oxepin	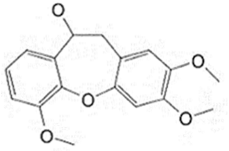	66.328	$C_{16}H_{16}O_4$	272.29	1.02	623334	*
33	Linoleoyl chloride	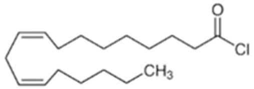	67.463	$C_{18}H_{31}ClO$	298.9	0.65	9817754	7459-33-8
34	Olealdehyde	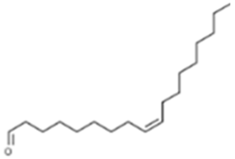	67.625	$C_{18}H_{34}O$	266.5	0.72	5364492	2423-10-1

35	Supraene	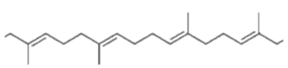	70.517	$C_{30}H_{50}$	410.7	0.66	638072	111-02-4
36	Abietinol	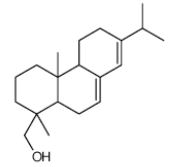	77.660	$C_{20}H_{32}O$	288.5	0.89	443474	666-84-2
37	cis-Valerenyl acetate	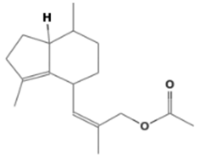	78.892	$C_{17}H_{26}O_2$	262.4	1.16	91730075	*
38	Stigmasterol	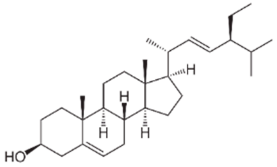	79.541	$C_{29}H_{48}O$	412.7	1.24	5280794	83-48-7
39	Bicyclo[4.4.0]dec-6-en-9.beta.-ol, 1,7-dimethyl-4.alpha.-isopropenyl	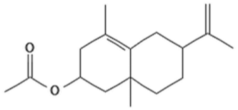	80.090	$C_{17}H_{26}O_2$	262.4	0.55	583165	*

40	Spiro[tricyclo[4.4.0.0(5,9)]decane-10,2'-oxirane], 1-methyl-4-isopropyl-7,8-dihydroxy-, (8S)-	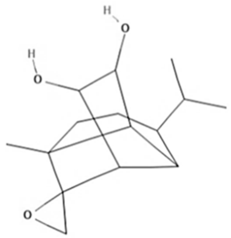	80.461	$C_{15}H_{24}O_3$	252.35	0.43	599405	*
41	2-(4a,8-Dimethyl-2,3,4,4a,5,6-hexahydro-naphthalen-2-yl)-prop-2-en-1-ol	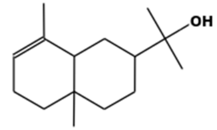	80.877	$C_{15}H_{22}O$	218.33	0.94	608286	*
42	Sitosterol	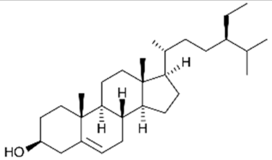	81.090	$C_{29}H_{50}O$	414.7	0.60	222284	83-46-5
43	2-(4a,8-Dimethyl-2,3,4,4a,5,6-hexahydro-naphthalen-2-yl)-prop-2-en-1-ol	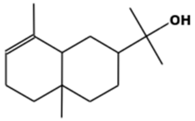	81.426	$C_{15}H_{22}O$	218.33	1.77	608286	*
44	cis-Valerenyl acetate	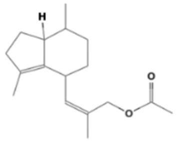	81.807	$C_{17}H_{26}O_2$	262.4	11.61	91730075	*

45	Betulin	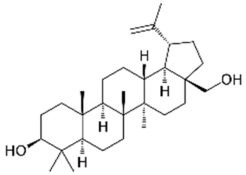	82.427	$C_{30}H_{50}O_2$	442.7	1.07	72326	473-98-3
46	Khusimyl methyl ether	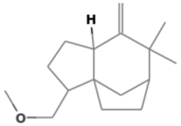	83.104	$C_{16}H_{26}O$	234.38	1.35	78409379	*
47	Longiborneol	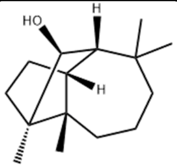	83.285	$C_{15}H_{26}O$	222.37	0.41	521200	*
48	Kolavenol acetate	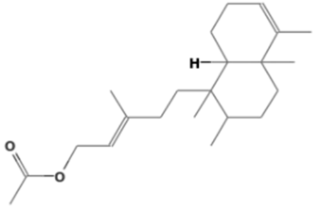	83.593	$C_{22}H_{36}O_2$	332.5	0.49	14137618	*

49	Phenylamine, 2-methyl-5-[5-(5-pentyl-[1,3]dioxan-2-yl)furan-2-yl]-	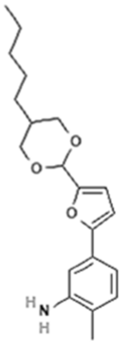	86.701	$C_{20}H_{27}NO_3$	329.4	5.53	6425198	*

Total = 99.51%

* not listed in the CAS database

Table S2. Elution gradient of the chromatographic analysis by HPLC.

Time (min)	Solvent A (%)	Solvent B (%)
0-5	100	0
5-10	70	30
10-20	60	40
20-60	60	40
60-70	50	50
70-90	40	60
90-100	20	80
100-110	0	100
110-120	0	100

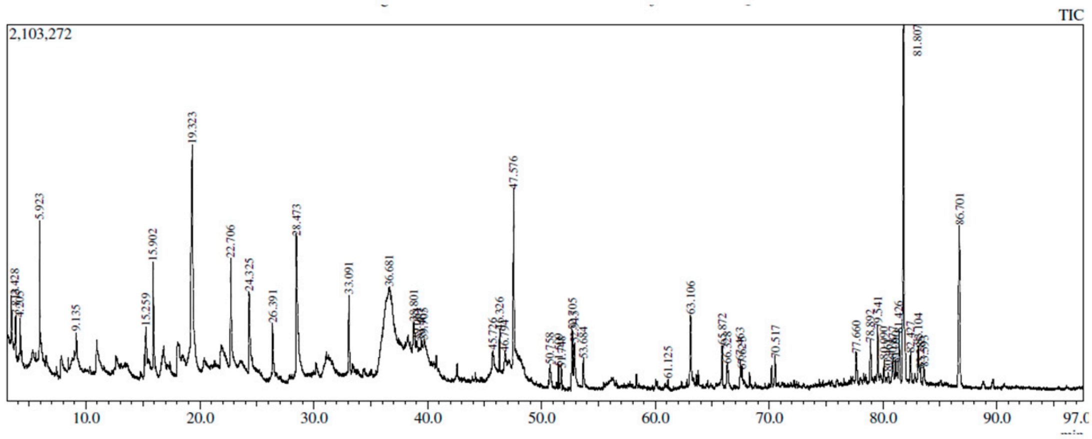

Figure S1. The fingerprint obtained by GC-MS of the methanolic extract obtained from *C. alba* roots by maceration (CAH21).

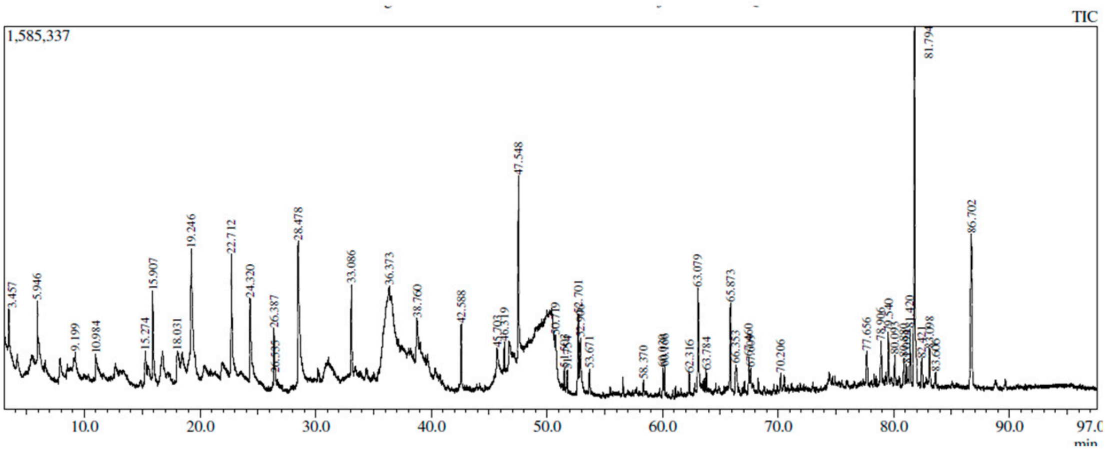

Figure S2. The fingerprint obtained by GC-MS of the methanolic extract obtained from *C. alba* roots by soxhlet (CAH24).

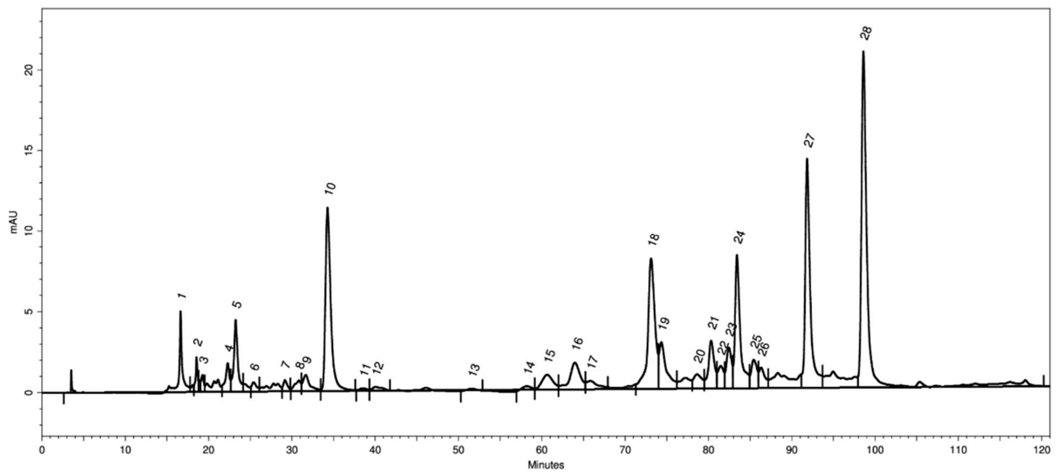

Figure S3. The fingerprint obtained by HPLC of the methanolic extract obtained from *C. alba* roots by maceration (CAH21). Peak 9: Naringin, detected at 280 nm.

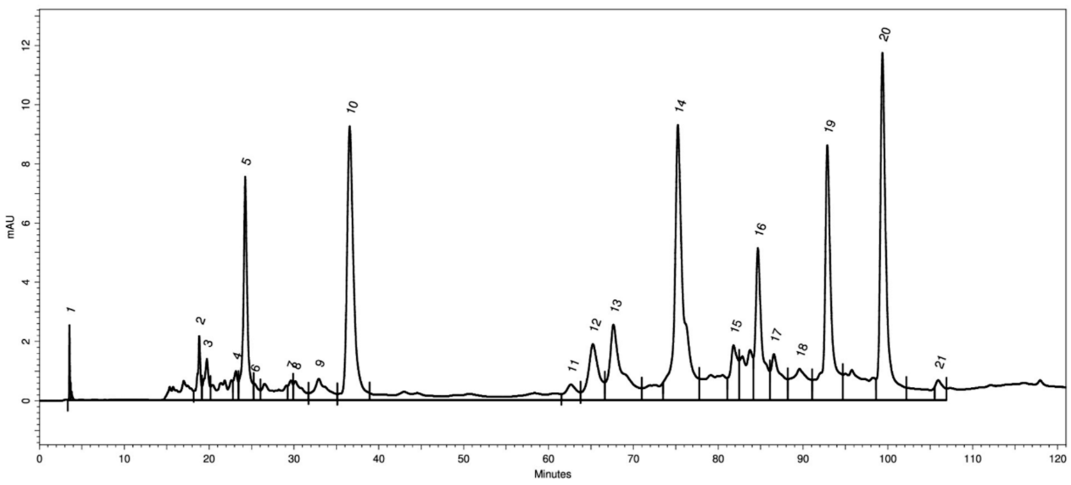

Figure S4. The fingerprint obtained by HPLC of the methanolic extract obtained from *C. alba* roots by soxhlet (CAH24). Peak 5: syringic acid, 6: chlorogenic acid, 10: vitexin, 12: myricetin, and 14: quercetin, detected at 280 nm.

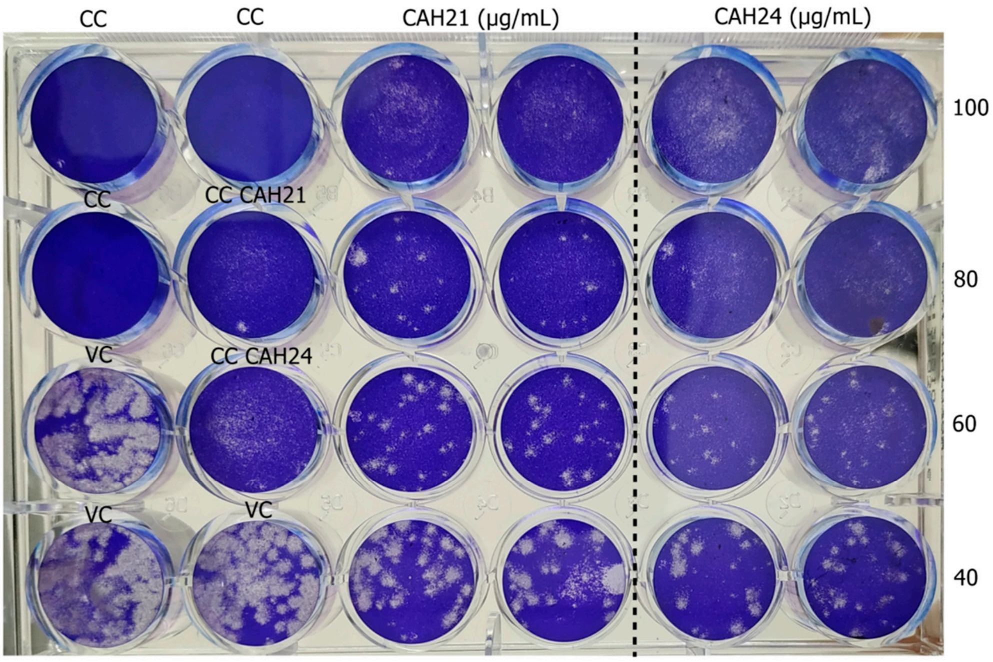

Figure S5. Plaque assay for CAH21 and CAH24 *C. alba* extracts against CHIKV on Vero cells monolayer for 48 hours. CC = cellular control; VC = viral control; CC CAH21 = cytotoxic control with 100 µg/mL of CAH21, and CC CAH24 = cytotoxic control with 100µg/mL of CAH24. Concentrations: 100 = 100 µg/mL; 80 = 80 µg/mL; 60 = 60 µg/mL, and 40 = 40 µg/mL.

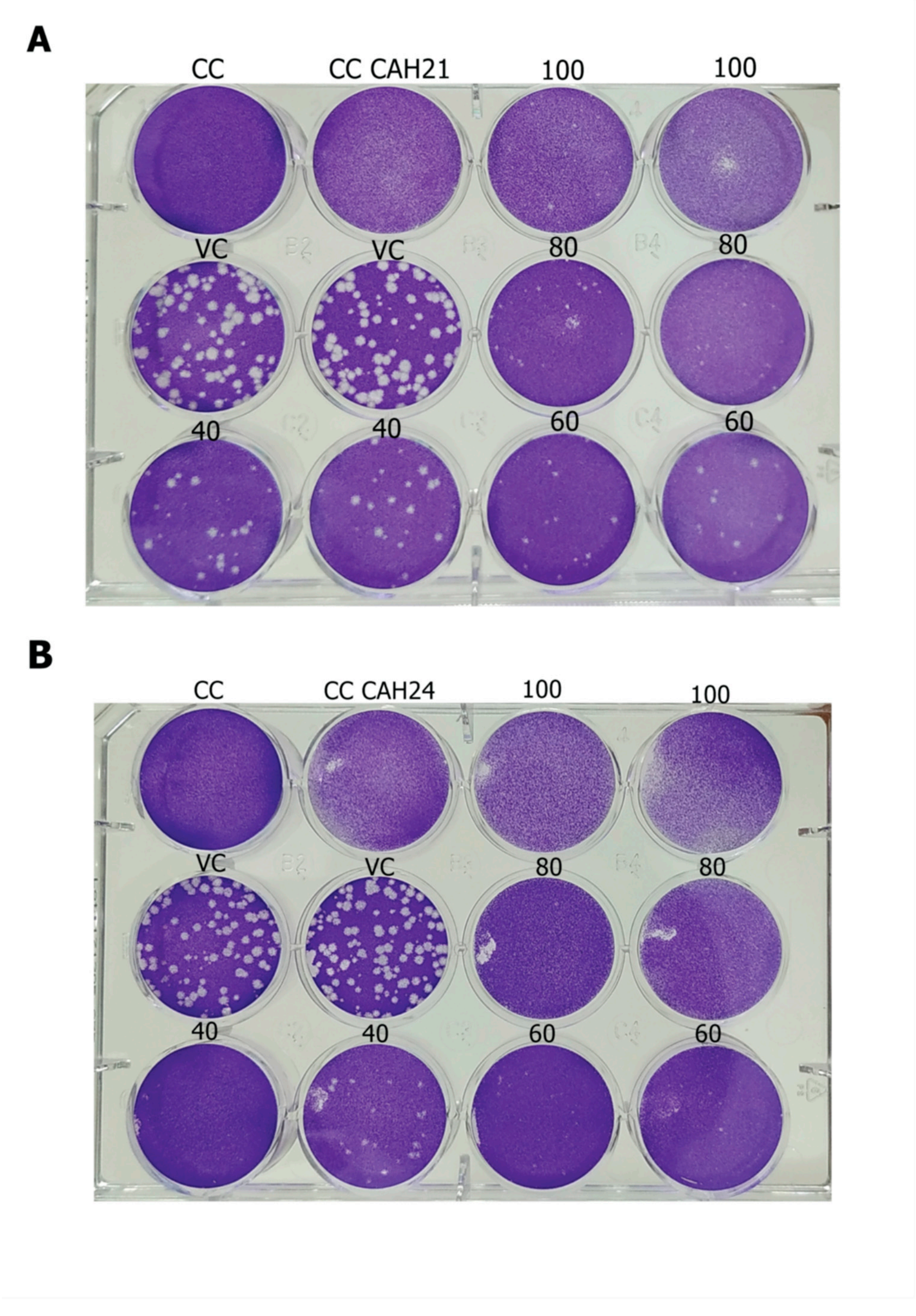

Figure S6. Plaque assay for CAH21 (A) and CAH24 (B) *C. alba* extracts against MAYV on Vero cells monolayer for 48 hours. CC = cellular control; VC = viral control; CC CAH21 = cytotoxic control with 100 µg/mL of CAH21, and CC CAH24 = cytotoxic control with 100µg/mL of CAH24. Concentrations: 100 = 100 µg/mL; 80 = 80 µg/mL; 60 = 60 µg/mL, and 40 = 40 µg/mL.

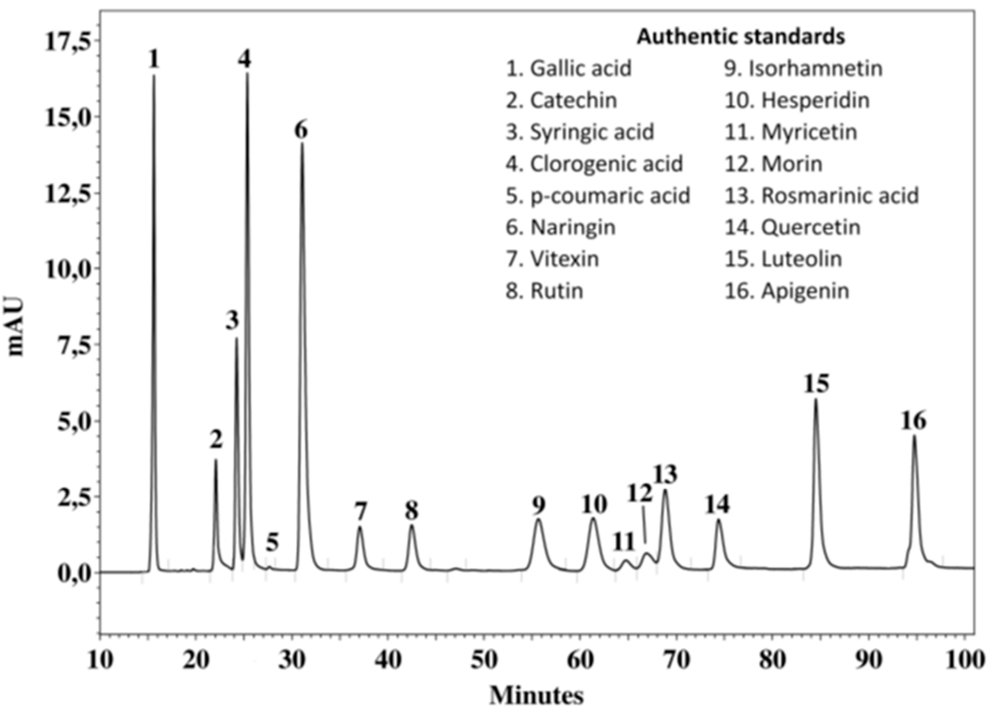

Figure S7. Authentic standards fingerprint available in High-Performance Liquid Chromatography (HPLC). Source: Laboratory of Scientific Instrumentation (LABIC), Federal University of Tocantins.
